# Editorial: New insights into social isolation and loneliness, volume II

**DOI:** 10.3389/fpsyt.2025.1675314

**Published:** 2025-08-14

**Authors:** Yuka Kotozaki

**Affiliations:** Department of Hygiene and Preventive Medicine, Iwate Medical University, Yahaba, Japan

**Keywords:** social isolation, loneliness, mental health, older adults, social support

This collection is the 2nd volume of the Research Topic, *New Insights into Social Isolation and Loneliness*. Social isolation is generally recognized as a major public health problem and is widely recognized to have detrimental consequences for people’s health, including reduced mental health, increased risk of disease (e.g., hypertension, cardiovascular disease, cancer), mortality, and cognitive decline. It is widely recognized that social isolation is a major cause of death. Combined with the recent COVID-19 and other changes in social conditions, social isolation, and loneliness are becoming an increasing concern. Social isolation can occur regardless of gender or age, and the occurrence process and related factors warrant further investigation.

The primary objective of this Research Topic is to deepen our understanding of social isolation and loneliness. A secondary objective is to explore their neurobiological underpinnings and provide insights that may inform the development and implementation of targeted prevention and intervention strategies for high-risk populations.

Out of the 24 manuscripts submitted by international researchers, 13 were accepted for publication following a rigorous peer review process. Below is a summary of the main findings from each of the accepted papers.

In the first article in this Research Topic, Worsley et al. examined the mental health of university students early in the COVID-19 pandemic and identified academic, non-academic, and COVID-19-related factors influencing depression and anxiety. Surveying 3,817 students, it found high rates of depression and anxiety (over 50%). Key risk factors included academic stress, social isolation, intolerance of uncertainty, and negative attitudes toward remote learning, while a strong sense of university belonging was protective. These findings offer valuable insights for developing targeted mental health support for students during future crises.

In the next article, Luo et al. conducted a systematic review and meta-analysis examining the link between living alone and suicidal behaviors using data from over 3.6 million participants across nine prospective studies. Their results showed that living alone was associated with a significantly higher risk of both suicide attempts and suicide death, even after adjusting for confounding factors. With increasing rates of living alone worldwide, these findings highlight the importance of addressing living arrangements in suicide prevention efforts.

Zhao and Kou investigated the relationship between loneliness and short video addiction among 388 college students, focusing on the mediating roles of social support and physical activity. Their results showed that loneliness directly increased short video addiction, with social support and physical activity independently mediating this effect. Additionally, social support and physical activity together formed a chain mediation between loneliness and addiction. These findings offer new insights and suggest effective strategies to reduce short video addiction among college students.

Meneguzzo et al. examined whether loneliness mediates the link between childhood trauma and eating disorder (ED) symptoms in 230 individuals with EDs. Their analysis showed that childhood trauma predicted higher loneliness, which in turn was associated with more severe ED symptoms. Loneliness partially mediated this relationship, suggesting that addressing loneliness in treatment could help reduce the impact of childhood trauma on ED severity. These findings highlight the potential of social connection–focused interventions in ED care.

Dong et al. investigated how social isolation mediates the relationship between stigma and work ability in young and middle-aged stroke patients. Their study found that higher stigma was associated with greater social isolation and reduced work ability. Social isolation partially mediated the negative impact of stigma on work ability, accounting for about 22% of the effect. These results highlight the importance of addressing both stigma and social isolation to improve work outcomes in stroke survivors.

Chen et al. developed a stacked ensemble machine learning model to predict Major Depressive Disorder (MDD) in adults living alone using health data from over 2,600 US participants. Their model showed strong accuracy (AUC = 0.85). Key factors positively associated with MDD included sleep disorders, medication use, and certain health conditions, while age and some dietary factors were negatively associated. This approach improves identification of MDD risk among adults living alone, aiding targeted mental health interventions.

Li et al. studied 4,819 middle-aged and older women who participated in square dance exercise to examine its impact on attitudes toward aging. Their analysis showed that square dance positively influenced aging attitudes, with loneliness reduction and improved quality of life acting as key mediators. Quality of life had the strongest mediating effect. These findings suggest that square dance exercise promotes positive aging attitudes and enhances well-being in this population.

Liu et al. studied 223 individuals with chronic schizophrenia to examine how psychotic symptoms affect social functioning. They found that impaired interpersonal trust mediates the relationship between psychotic symptoms and reduced social functioning. These results suggest that interventions aimed at improving interpersonal trust could enhance social functioning and outcomes in schizophrenia patients.

Mohammed Ali et al. validated the Three-Item UCLA Loneliness Scale (UCLALS3) for dementia family caregivers (N=571). The scale showed strong reliability and validity, with a cutoff score of ≥6.5 effectively identifying caregivers experiencing high loneliness linked to greater caregiving burden and distress. This short measure can help detect caregivers in need of targeted social support interventions.

Meehan et al. conducted a 12-year longitudinal study in Australia examining community-level factors influencing loneliness and social isolation (SI) among younger (18–30) and older (60+) adults. They found that low community engagement was the strongest risk factor for both loneliness and SI across all ages. Additionally, low neighborhood social cohesion significantly increased loneliness and SI, especially in older adults. The study emphasizes the importance of community-focused interventions to effectively reduce loneliness and social isolation beyond individual-level approaches.

Ju et al. studied loneliness in stroke patients aged 60 and older, finding moderate levels of loneliness influenced by income, activity, community resources, social participation, and illness duration. Family resilience directly reduced loneliness, and this effect was partially mediated by social support, family functioning, and psychological capital. The findings suggest that enhancing family resilience and support can effectively alleviate loneliness in elderly stroke survivors.

Wenig et al. conducted a large cross-sectional study of 12,874 German university students to assess loneliness and its associated factors. They found that 28.2% of students experienced loneliness, with higher risk among gender-diverse individuals, males, and those with poor health. Positive social relationships and strong university connectedness reduced loneliness, while weak ties increased it. Students at universities of applied sciences reported less loneliness. The study highlights the need for individual and institutional efforts to foster social connections and community engagement to combat loneliness in universities.

Finally, Gorman et al. examined how the Veterans Socials program, a community-based social support initiative for U.S. veterans, transitioned from in-person to virtual gatherings during COVID-19. The study highlights how partnerships between private and government organizations enabled the program to maintain social connections, reduce isolation, and improve healthcare access during the pandemic. Their findings emphasize the critical role of community collaboration in sustaining support and disseminating vital information during emergencies.

In conclusion, this Research Topic provides comprehensive and multi-faceted insights into the complex issue of social isolation and loneliness across diverse populations and contexts. Spanning different age groups—from university students and middle-aged adults to elderly stroke survivors and dementia caregivers—and geographic regions, the research highlights both common and unique risk factors, including individual, interpersonal, and community-level determinants.

Key findings emphasize the critical role of social support, family resilience, and community engagement in mitigating loneliness and its adverse mental and physical health consequences. Several studies underline the importance of targeted interventions tailored to specific populations, such as trust-building in schizophrenia patients, physical activity programs for older women, and technology-facilitated social connection for veterans.

Moreover, the integration of neurobiological, psychological, and social ecological perspectives enriches our understanding of the mechanisms driving loneliness and social isolation. This knowledge informs innovative approaches, including predictive modeling and mediation analyses, which can guide effective prevention and treatment strategies.

Overall, this Research Topic reinforces that addressing social isolation and loneliness requires holistic, multi-level strategies that consider individual vulnerabilities alongside broader social and environmental factors. As social dynamics continue to evolve, especially under challenges like the COVID-19 pandemic, these findings offer valuable guidance for policymakers, healthcare providers, and communities aiming to promote social inclusion and improve well-being.

